# Patterns of tobacco use and related factors among adolescents in South Africa: Insight from the Global Youth Tobacco Survey

**DOI:** 10.18332/tid/207126

**Published:** 2025-07-26

**Authors:** Mukhethwa Londani, Constance Sewani-Rusike, Olalekan Ayo-Yusuf, Olanrewaju Oladimeji

**Affiliations:** 1 Directorate of Research and Innovation, Tshwane University of Technology, Pretoria, South Africa; 2 Department of Human Biology, Faculty of Medicine and Health Sciences, Walter Sisulu University, Mthatha, South Africa; 3 Africa Centre for Tobacco Industry Monitoring and Policy Research, School of Health Systems and Public Health, University of Pretoria, Pretoria, South Africa; 4 Department of Epidemiology and Biostatistics, Sefako Makgatho Health Sciences University, Pretoria, South Africa; 5 Department of Social Sciences, Demography and Population Studies Unit, Walter Sisulu University, Mthatha, South Africa

**Keywords:** adolescents, multiple tobacco products, combustible noncigarette tobacco products, South Africa

## Abstract

**INTRODUCTION:**

Adolescent multiple tobacco use is a major public health issue, both in South Africa and globally. This study sought to use latent class analysis to identify patterns of tobacco products use (combustible cigarettes, chewing tobacco, snuff) and combustible non-cigarette tobacco products smoking (such as cigars, little cigars, pipes) and related factors among adolescents in South Africa.

**METHODS:**

Data from the Global Youth Tobacco Survey (2011) were used. A nationally representative cross-sectional school-based survey was conducted among secondary school students using a stratified two-stage cluster sampling. The sample comprised 10822 students in grades 8 to 11 in South Africa (approximately aged 13–18 years). Latent class analysis was used to identify patterns of tobacco use and examined how sociodemographic and tobacco-related characteristics are associated with such patterns. Subsequently, the multinomial logistic regression analysis was used to assess the relationship between covariates and tobacco-related variables with the probability of belonging to a specific latent class.

**RESULTS:**

The latent class analysis identified three classes: Class 1 (8.8%) was characterized by high probabilities of multiple tobacco product use; Class 2 (3.4%) had high probabilities of combustible non-cigarette tobacco products smoking; and Class 3 (83.9%) showed a minimal likelihood of current use across all four products. Compared to non-users, adolescents exposed to peer smoking had significantly higher odds of multiple product use (AOR=4.07; 95% CI: 2.93–5.66) and combustible tobacco use (AOR=6.29; 95% CI: 4.15–9.53). Parental smoking was also associated with increased odds of both multiple (AOR=2.33; 95% CI: 1.81–3.00) and combustible (AOR=1.91; 95% CI: 1.31–2.79) tobacco use. Females had lower odds than males of using multiple (AOR=0.65; 95% CI: 0.49–0.87) and combustible products (AOR=0.51; 95% CI: 0.36–0.71). Older adolescents (aged ≥18 years) were more likely to be multiple users (AOR=4.18; 95% CI: 1.59–10.98). Support for smoke-free policies was associated with reduced odds of tobacco use, while knowledge of smoking harms was associated with combustible tobacco use (AOR=1.60; 95% CI: 1.07–2.39).

**CONCLUSIONS:**

Multiple tobacco use and combustible non-cigarette tobacco products smoking is common among South African adolescents. Understanding different patterns of multiple tobacco use and combustible non-cigarette tobacco products smoking can help to inform prevention and cessation programs for adolescents. Given the risk adolescents face, tobacco cessation interventions tailored to their tobacco product of choice are urgently needed.

## INTRODUCTION

Tobacco use remains a major public health challenge, urging countries to seek more effective strategies to reduce prevalence and address the related health risks^1^. The World Health Organization (WHO) estimates that around 1.25 billion adults use tobacco globally, which includes both smoked and smokeless products^2^. Alarmingly, approximately 24 million adolescents aged 13–15 years also use tobacco, showing a concerning trend of early initiation.

Tobacco use among adolescents and young adults represents a complex phenomenon characterized by a developmental trajectory that includes experimentation, occasional use, dependence, regular use, and, for some, eventual cessation^3^. Globally, there are approximately 1.8 billion adolescents and young adults^4^, with Sub-Saharan Africa being the only region where the mortality rate for individuals aged ≤24 years has increased since 1950^5^. Mental health challenges, including substance use disorders, contribute to approximately 55.5 million disability-adjusted life years (DALYs) among adolescents and young adults globally, accounting for up to 10% of all DALYs in Southern Africa^6^.

In South Africa, 25.3% of the population is aged 10–24 years^7^. Tobacco use within this group has been a significant public health concern, influenced by factors such as peer pressure, exposure to tobacco advertising, socio-economic status, and cultural norms^8^. Efforts have been made to curb smoking through legislation, education, and anti-smoking campaigns, but challenges related to reducing smoking prevalence in South Africa remain.

Tobacco use is a primary contributor to preventable diseases and deaths, with significant health consequences such as cardiovascular disease, cancer, respiratory diseases, and various other chronic conditions^1^. Concerns have expanded beyond immediate health risks to include the long-term impact of lifetime exposure to tobacco smoke, particularly in terms of intergenerational health effects^9^. In addition, concerns have been raised regarding adolescents experiencing negative health consequences and an increasing burden on health and social services^2^.

Tobacco use during adolescence and early adulthood can result in structural and functional alterations in the brain, affecting specific brain regions and the endocannabinoid system^10^. Cognitive impairments such as on IQ and verbal learning may persist into adulthood with continued tobacco use, and adolescents may be more susceptible to substance use disorders^11^. Although the relationship between schizophrenia and adolescent tobacco use remains unclear, tobacco use among adolescents is considered an increased risk of having psychosis, depression and suicidal behaviour^12^.

Tobacco use is notably prevalent among people with psychiatric diagnoses, including specific conditions such as attention deficit hyperactivity disorder (ADHD), anxiety disorders, and substance abuse^13^. Daily smoking among adolescents and young adults was associated with a 70% increase in the likelihood of being diagnosed with anxiety, mood disorders and disruptive behavior disorders^14^. Moreover, psychiatric comorbidity is common in adolescent cigarette smokers, particularly in individuals with disruptive behavior disorders (such as oppositional defiant disorder, conduct disorder, and ADHD), major depressive disorders, and drug and alcohol use^15^. Adolescents are considered more vulnerable to the negative effects of nicotine dependence than adults^16^. Thus, early initiation of cigarette smoking (before the age of 13 years) and the early onset of conduct problems are significant indicators of increased psychopathology in later life^17^. Despite global initiatives aimed at reducing tobacco use through public health campaigns, smoking bans, and increased taxes on tobacco products, these efforts face significant challenges due to the ongoing increase in tobacco use, especially in low- and middle-income countries^2^.

Polytobacco or multiple tobacco product use is common among adolescents and represents a significant public health concern^18^. Research consistently indicates that adolescents are increasingly using a combination of tobacco products, including cigarettes, smokeless tobacco, and cigars^19^. This behavior is associated with increased risks of nicotine dependence and various health challenges. In South Africa, although national surveys and interventions often focus on cigarette smoking, adolescents are increasingly experimenting with a variety of tobacco products. Yet, most research has treated tobacco use as a uniform behavior, overlooking the complexities and risks associated with multiple-product use. This limited approach potentially weakens the effectiveness of tobacco control efforts. In line with this, the aim of this study is to assess, using latent class analysis, heterogeneity in underlying patterns of tobacco use based on four types of tobacco products (combustible cigarettes, chewing tobacco, snuff and combustible non-cigarette tobacco products smoking (such as cigars, little cigars, pipes) among adolescents in South Africa, and to examine how sociodemographic and tobacco-related characteristics are associated with these underlying patterns of tobacco use.

## METHODS

### Study design and sampling method

This study used data collected from South Africa’s Global Youth Tobacco Survey (GYTS). The 2011 nationally and provincially representative cross-sectional school-based survey was conducted among secondary school students using the GYTS study protocols established by the US Centers for Disease Control and Prevention (CDC). The GYTS is part of the Global Tobacco Surveillance System that enhances countries’ ability to design, implement, and assess tobacco control interventions. The GYTS used a two-stage cluster sampling design, stratified by the nine provinces of the country, to provide data that are representative at both national and provincial levels.

In the initial stage of sampling, schools served as the primary sampling units and were chosen based on a probability proportional to the enrolment size of students in grades 8 to 11. The national databases of all public schools with grades 8 to 11 were acquired from the National Department of Education (DoE) and served as the sampling frames for the initial stage of sampling. Private schools, constituting 4.4% of all educational institutions, were omitted due to logistical considerations^20^. A total of 10963 public schools, with a combined enrolment of 3.7 million students in grades 8 to 11, were eligible for selection^20^.

In the second stage of sampling, classes from grades 8 to 11 were selected from each participating school through systematic equal probability sampling with a random initiation. All students in the chosen classes were eligible to participate. Twenty-three schools were selected per province, with an average of two classes per school. The GYTS employed a standardized methodology for developing sampling frames, selecting schools and classes, preparing questionnaires, executing field procedures, and processing data^2^. The collected data were weighted to account for complex sample design and sample frame. The overall response rate was 69.1%.

### Data collection

The GYTS survey used a standardized, self-administered questionnaire, provided by the CDC. The standard English questionnaire was adapted for use in South Africa. The GYTS questionnaire was translated into ten South African official languages to meet the linguistic requirements and preferences of all students (sign language had not yet been recognized as an official language at the time of the survey). The ten languages are Sepedi, Sesotho, Setswana, siSwati, Tshivenda, Xitsonga, Afrikaans, isiNdebele, isiXhosa, and isiZulu. Furthermore, back translation was conducted to ensure the elimination of any bias resulting from the translation of the materials. This process involved using a proficient speaker of an indigenous language, who had no previous familiarity with the original English version, to translate the questionnaire back into English. The back translation was then compared to the original version, and any discrepancies were analyzed and corrected as necessary.

The final questionnaire comprised 56 closed-ended questions including seven domains associated with tobacco use (both smoking and smokeless), cessation, secondhand smoke, pro- and anti-tobacco media and advertising exposure, access to and availability of tobacco products, and knowledge and attitudes regarding tobacco, specifically being taught in school about the harmful effects of tobacco use.

### Measures

This section provides a detailed description of the pertinent questions and variables used for the analysis.

*Latent class indicators*

Four tobacco use variables were included in the latent class model. Adolescents were asked ‘During the past 30 days, on how many days did you smoke or use the following?’ (number of days) for combustible cigarettes, smokeless tobacco (chewing tobacco, snuff) and combustible non-cigarette tobacco products smoking (such as cigars, little cigars, pipes). These variables were dichotomized into current use (1–30 days) and non-current use (0 days).

*Sociodemographic characteristics*

Characteristics included: sex (male, female); age (≤13, 14, 15, 16, 17, or ≥18 years) and grade (8, 9, 10, or 11).

*Tobacco advertising*

Exposure to tobacco advertising was measured by combining five sources of advertising that adolescents indicated they had encountered for tobacco products. Adolescents were categorized as having been exposed to tobacco advertisements if they selected ‘A lot’, ‘Sometimes’, ‘A lot’ or ‘A few’ to any of the following questions: ‘During the past 30 days (one month), when you watched sports events or other programs on TV how often did you see cigarette brand names?’; ‘During the past 30 days (one month), how often did you hear cigarette brand names mentioned when you listened to the radio?’; ‘When you go to sports events, fairs, concerts, or community events, how often do you see advertisements for cigarettes?’; ‘During the past 30 days (one month), how many advertisements for cigarettes have you seen on billboards?’; and ‘During the past 30 days (one month), how many advertisements or promotions for cigarettes have you seen in newspapers or magazines?’.

*School curriculum*

The adolescents who answered ‘Yes’ to any of the following questions: ‘During this school year, were you taught in any of your classes about the dangers of smoking?’; ‘During this school year, were you taught in any of your classes that most people your age do not smoke cigarettes?; and ‘During this school year, were you taught in any of your classes about the effects of smoking (such as it makes your teeth yellow, causes wrinkles, or makes you smell bad)?’ were categorized as receiving an educational curriculum regarding the danger of smoking.

*Peer smoking*

Adolescents were categorized as 0 if none of their closest friends smoked and 1 if some or all their closest friends smoked.

*Parental/guardians smoking*

Adolescents were categorized as 0 if neither parent/guardian smoked and as 1 if either or both parents/guardians smoked.

*Support for smoke-free policy*

Adolescents were asked: ‘Are you in favor of banning (not allowing) smoking in public places (such as in restaurants, in buses and trains, in schools, on playgrounds, in gyms and sports arenas, in discos/clubs)?’. Adolescents were categorized as 0 if they answered ‘No’ and as 1 if they answered ‘Yes’.

*Knowledge about the harm of smoking*

Adolescents were asked: ‘Do you think cigarette smoking is harmful to your health?’. Adolescents were categorized as 0 if they answered ‘Definitely not’ and as 1 if they answered ‘Probably not’, ‘Probably yes’, or ‘Definitely yes’.

### Ethics procedures

The South African Medical Research Council’s Research Ethics Committee approved the survey’s protocol, measures, and procedures (MRC GYTS 12/18/2010). The procedures were designed for voluntary participation, and confidentiality was maintained throughout the data collection. The study was conducted by asking students in selected classes to complete questionnaires, and the entire process was overseen by trained researchers. The National Department of Education, school principals, students’ parents or guardians, and students provided informed consent forms^20^. On the day of the survey, students aged <18 years also provided their assents. The data were downloaded and accessed for the purposes of the present study on 17 May 2022.

### Statistical analysis

Data were analyzed using Mplus 8.2 and STATA version 17, StataCorp, College Station, TX, USA. The data were weighted to account for complex multi-stage sampling design. Prior to conducting latent class models, survey-adjusted descriptive analyses were performed. A series of independently estimated latent class analyses were used to classify students’ groups based on their tobacco use patterns. Four dichotomous variables were used to define the latent classes, and conditional probabilities were applied to assign participants to these classes.

The goodness of fit indices was assessed by examining the overall model fit, which included the Akaike information criterion (AIC), Bayesian information criterion (BIC), Sample-size adjusted Bayesian information criterion (ABIC), Lo-Mendell-Rubin likelihood ratio test (LMRT), and entropy. The most frequently reported fit statistic is the BIC, with lower values indicating a good fit. The BIC rewards model parsimony when the value is low (indicating a high log likelihood estimate and a low number of parameters), with differences of ≥10 regarded as evidence supporting one model over another. Additionally, an entropy summary statistic assessed the classification quality. This statistic ranges from 0 to 1, with values approaching 1 indicating superior classification quality. The entropy value for the selected class was 0.85. The Lo-Mendell-Rubin or bootstrapped likelihood ratio tests were used to assess the likelihood that the data can be represented by a model with one-less class and a p<0.05 indicating that the inclusion of the additional class substantially improves the fit over a model with k-1 classes. After identifying the best class model, posterior class membership probabilities were used to assign participants to classes. One fundamental assumption of LCA is that measurement error generates a degree of homogeneous, mutually exclusive error, assuming the measurement invariance of latent classes. To ascertain the adequate number of classes, an initial single-class model was estimated, followed by the sequential addition of classes until the best fitting model was identified. Each individual was assigned to the most probable class according to the highest probability derived from the retained latent class model. Once the number of classes was determined, the final model was estimated with sociodemographic (sex, age, and grade) and tobacco related variables (tobacco advertising, school curriculum, peer smoking, parents/guardians smoking, support for smoke-free policy, knowledge about the harm of smoking) covariates using the R3STEP command in Mplus.

The three-step method was used to conduct multinomial logistic regression analyses. Odds ratios derived from the multinomial logistic regression analysis show the relationship between covariates and tobacco-related variables with the probability of belonging to a specific latent class. Missing data on tobacco use variables were excluded from the analysis.

The total sample of students in the GYTS study was 10833. Eleven adolescents failed to report their tobacco use status across all four variables. The analysis excluded these adolescents. The analysis comprised a final sample size of 10822 students.

## RESULTS

### Sample characteristics of participants

As shown in Table 1, slightly more than half (51.2%) of the adolescents were female, with the largest group (22.4%) aged ≥18 years. Majority of the participants were in grade 10 (26.8%), followed by those in grade 8 (26.0%). In terms of tobacco-related variables, the majority (88%) of participants were exposed to tobacco advertising, about 72% were taught about the dangers of smoking, and most participants (58%) supported a 100% smoke-free policy. Less than half of the participants had peers (40.7%) and/or parents/guardians (31.4%), respectively, smoking around them. A higher proportion of the participants were aware of the harms of smoking (77.1%). In our study, 17.9% were combustible cigarette smokers, 14.3% used smokeless tobacco (7.3% used chewing tobacco and 7.0% used snuff) and 19.0% smoked combustible non-cigarette tobacco products such as cigars, little cigars, and pipes, in the past 30 days.

**Table 1:** Sociodemographic characteristics, tobacco-related social exposure and awareness factors, and tobacco use status of adolescents in grades 8–11, a cross-sectional school-based survey, South Africa, GYTS 2011 (N=10822)

*Characteristics*	*Unweighted n* *(Weighted %)*
**Gender**	
Male	5213 (48.8)
Female	5499 (51.2)
**Age** (years)	
≤13	710 (6.4)
14	1396 (14.5)
15	2054 (18.3)
16	2182 (19.0)
17	2039 (19.4)
≥18	2370 (22.4)
**Grade**	
8	2586 (26.0)
9	2894 (24.1)
10	3015 (26.8)
11	2167 (23.1)
**Tobacco advertising**	
No	1159 (11.6)
Yes	9617 (88.4)
**School curriculum**	
No	2741 (27.9)
Yes	7402 (72.1)
**Peer smoking**	
No	6225 (59.3)
Yes	4383 (40.7)
**Parents/guardians smoking**	
No	6508 (68.6)
Yes	3413 (31.4)
**Support for smoke-free policy**	
No	4440 (41.6)
Yes	6037 (58.4)
**Knowledge about the harm of smoking**	
No	2764 (22.9)
Yes	7889 (77.1)
**Combustible cigarettes**	
No	8168 (82.1)
Yes	1855 (17.9)
**Chewing tobacco**	
No	9839 (92.7)
Yes	819 (7.3)
**Snuff**	
No	10012 (93.0)
Yes	758 (7.0)
**Combustible non-cigarette tobacco**	
No	8361 (81.0)
Yes	2230 (19.0)

### Identify latent classes of tobacco use

The model fit was assessed for models comprising between 1 and 4 class. The optimal fit was attained with three classes, identified as the best fit of the latent class model using bootstrapped likelihood ratio test (BLRT), LMRT, BIC, ABIC (which performs effectively for categorical data), AIC^21^ and entropy (Table 2). All information criteria dropped from the one-class to the three-class models. Entropy was determined to be satisfactory (0.849), and the LMRT test was likewise statistically significant at class three. The three-class solution revealed the lowest values relative to the other classes. Class 1 members, characterized by current multiple tobacco product use, constituted 8.8% of the sample. In this class, participants showed a 76% probability of combustible cigarette smoking, 80% for chewing tobacco and 55% of current users of snuff, and 44% probability of using combustible non-cigarette tobacco products. Class 2 members are adolescents who had 100% of non-cigarette tobacco product smoking and 33% of cigarettes use, and accounted for 3.4% of the sample. Lastly, participants in class 3 (non-tobacco use) accounted for 83.9% of the sample and showed a minimal probability of current use across all four products (Table 3).

**Table 2:** Fit indices for multilevel latent classes of adolescents’ tobacco use status using dichotomous indicators, a cross-sectional school-based survey, South Africa, GYTS 2011 (N=10822)

*Class*	*AIC*	*BIC*	*ABIC*	*Entropy*	*LMRT*	*BLRT*	*Size*
1	31773.32	31802.47	31789.76	-	-	-	10822
2	28943.91	29009.51	28980.91	0.908	<0.001	<0.001	9869 | 953
3	**28892.54**	**28994.59**	**28950.10**	**0.849**	**<0.001**	**<0.001**	**953 | 369 | 9500**
4	28900.28	29038.77	28978.39	0.662	0.743	1.000	8344 | 852 | 194 | 1432

AIC: Akaike information criterion. BIC: Bayesian information criterion. ABIC: Sample-size adjusted Bayesian information criterion. LMRT: Lo-Mendell-Rubin likelihood ratio test. BLRT: bootstrapped likelihood ratio test.

**Table 3:** Conditional probabilities of tobacco use among adolescents, a cross-sectional school-based survey, South Africa, GYTS 2011 (N=10822)

*Tobacco use*	*Multiple tobacco product use*	*Combustible non-cigarette tobacco smoking*	*Non-tobacco use*
**Combustible cigarettes**			
No	0.240	0.669	0.876
Yes	0.760	0.331	0.124
**Chewing tobacco**			
No	0.198	1.000	0.999
Yes	0.802	0.000	0.001
**Snuff**			
No	0.449	1.000	0.979
Yes	0.551	0.000	0.021
**Combustible non-cigarette tobacco**			
No	0.556	0.000	0.874
Yes	0.444	1.000	0.126

### Latent profiles of tobacco use

**Figure 1:**
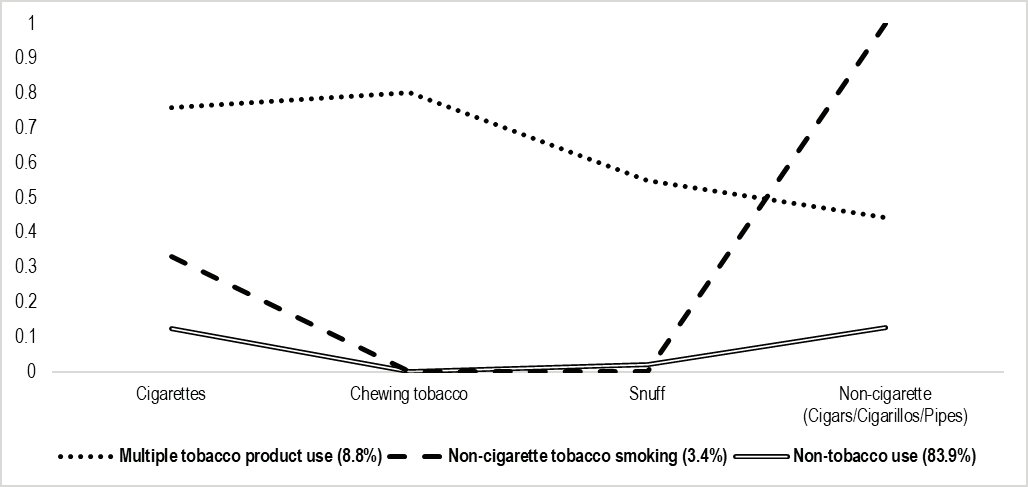
Latent profiles of tobacco use among adolescents, a cross-sectional school-based survey, South Africa, GYTS 2011 (N=10822)

Figure 1 illustrates a graphical representation of the three-class model. The x-axis enumerates the tobacco use variables. The y-axis represents the average probability of class membership for each variable; as the value approaches 1, the likelihood of class membership is higher. Additionally, Figure 1 illustrates the characteristics of the three classes based on responses to the four variables. A predominant proportion of participants (83.9%) belonged to the non-tobacco use category. Conversely, small percentages of the sample (8.8% and 3.4%) were in the multiple tobacco product use and combustible non-cigarette tobacco smoking classes, respectively.

### Prevalence of tobacco use by sociodemographics and tobacco-related variables

Bivariate chi-squared analyses were performed to investigate individual variables as possible correlates of class membership (Table 4). The bivariate analysis showed differences in class composition across all examined variables, except for grade which showed no statistical difference by classes. Males had a higher percentage of multiple tobacco product use (10.7%) and combustible non-cigarette tobacco products smoking (4.7%) than females. The percentage of multiple tobacco product use (11.1%) and the smoking of combustible non-cigarette tobacco products (4.0%) was significantly higher among adolescents aged ≥18 years. In addition, adolescents exposed to tobacco advertising had a greater prevalence of multiple tobacco product use (8.7%) and combustible non-cigarette tobacco products smoking (3.4%) compared to the non-exposed adolescents. Adolescents who were educated about the dangers of smoking and its effects had lower percentage of multiple tobacco product use (7.6%) and higher percentage of combustible non-cigarette tobacco products smoking (3.7%) compared those who were not educated. Adolescents exposed to smoking by peers and/or parents/guardians had multiple tobacco product use of 14.9% and 12.0%, and the smoke of combustible non-cigarette tobacco products at 6.7% and 5.2%, respectively. Adolescents in support of the smoke-free policy had less proportion of multiple tobacco product use (6.0%) and combustible non-cigarette tobacco products smoking (2.8%) compared to adolescents who opposed the policy. Lastly, knowing the harms of smoking (7.9%) was associated with a decreased likelihood of belonging to the multiple tobacco product use class, while concurrently increasing the probability of being grouped within the combustible non-cigarette tobacco products smoking category (3.7%).

**Table 4:** Prevalence of tobacco use by sociodemographics and tobacco-related variables, a cross-sectional school-based survey, South Africa, GYTS 2011 (N=10822)

*Variables*	*Multiple tobacco product use* *n (%)*	*Combustible non-cigarette tobacco products* *smoking n (%)*	*Non-tobacco use* *n (%)*	p*
**Overall**	953 (8.8)	369 (3.4)	9500 (83.9)	
**Sex**				**<0.001**
Male	619 (10.7)	259 (4.7)	4335 (84.6)	
Female	299 (5.6)	104 (1.9)	5096 (92.5)	
**Age** (years)				**0.002**
≤13	107 (10.1)	13 (1.0)	590 (88.9)	
14	75 (4.2)	33 (2.6)	1288 (93.2)	
15	115 (6.4)	64 (3.0)	1875 (90.6)	
16	171 (8.9)	63 (3.0)	1948 (88.1)	
17	180 (8.4)	80 (4.1)	1779 (87.5)	
≥18	285 (11.1)	112 (4.0)	1973 (85.0)	
**Grade**				0.114
8	236 (8.5)	54 (1.9)	2296 (89.6)	
9	261 (10.1)	92 (3.4)	2541 (86.5)	
10	247 (7.0)	121 (3.7)	2647 (89.3)	
11	159 (6.5)	93 (4.0)	1915 (89.5)	
**Tobacco advertising**				**<0.001**
No	54 (4.6)	27 (1.9)	1078 (93.6)	
Yes	870 (8.7)	340 (3.4)	8407 (87.9)	
**School curriculum**				**0.002**
No	300 (10.1)	77 (2.2)	2364 (87.7)	
Yes	596 (7.6)	276 (3.7)	6530 (88.7)	
**Peer smoking**				**<0.001**
No	219 (3.4)	65 (0.9)	5941 (95.7)	
Yes	659 (14.9)	298 (6.7)	3426 (78.4)	
**Parents/guardians smoking**				**<0.001**
No	371 (5.0)	180 (2.6)	5957 (92.5)	
Yes	393 (12.0)	165 (5.2)	2855 (82.8)	
**Support for smoke-free policy**				**<0.001**
No	389 (9.3)	147 (3.6)	3904 (87.1)	
Yes	406 (6.0)	189 (2.8)	5442 (91.2)	
**Knowledge about the harm of smoking**				**0.007**
No	220 (8.3)	53 (1.8)	2491 (89.8)	
Yes	688 (7.9)	315 (3.7)	6886 (88.4)	

* Significant at p<0.05 (chi-squared test). All data are weighted.

### Multinomial logistic regression predicting tobacco use

Multinomial logistic regression was performed to investigate the relative influence of potential correlates of class membership (Table 5). Non-tobacco use was used as baseline category for the outcome variable. Also, first categories in all the predictor variables were regarded as the baseline reference and the results were interpreted accordingly. As opposed to non-tobacco users, females had lower odds of being multiple tobacco product users (AOR=0.65; 95% CI: 0.49–0.87; p=0.004) and combustible non-cigarette tobacco products smokers (AOR=0.51; 95% CI: 0.36–0.71; p<0.001) compared to males. In addition, adolescents aged ≥18 years (AOR=4.18; 95% CI: 1.59–10.98; p=0.004) had more than four times odds of being multiple tobacco product users compared to non-tobacco users, in contrast to those aged ≤13 years. In comparison to adolescents in grade 8, those in grade 10 (AOR=0.29; 95% CI: 0.14–0.59; p=0.001) and grade 11 (AOR=0.22; 95% CI: 0.12–0.43; p<0.001) had lower odds of being multiple tobacco product users as opposed to non-tobacco users. Adolescents exposed to tobacco advertising had higher odds of smoking combustible non-cigarette tobacco products (AOR=1.70; 95% CI: 0.19–2.43; p=0.001) compared to the non-exposed adolescents. In contrast to non-tobacco users, adolescents exposed to peer smoking were over 4 times more likely to be multiple tobacco product users (AOR=4.07; 95% CI: 2.93–5.66; p<0.001) and more than six times more likely to be combustible non-cigarette tobacco products smokers (AOR=6.29; 95% CI: 4.15–9.53) compared to the non-exposed adolescents. Additionally, adolescents exposed to parents or guardian smoking were more likely to be multiple tobacco product users (AOR=2.33; 95% CI: 1.81–3.00; p<0.001) and combustible non-cigarette tobacco smokers (AOR=1.91; 95% CI: 1.31–2.79; p=0.001) compared non-tobacco users. Adolescents supporting smoke-free policies had lower odds of being multiple tobacco product users (AOR=0.73; 95% CI: 0.58–0.92; p=0.009) and combustible non-cigarette tobacco smokers (AOR=0.61; 95% CI: 0.41–0.91), rather than non-tobacco users, compared to those who did not support such policies. Lastly, knowledge about the harm of smoking was associated with a higher likelihood of smoking combustible non-cigarette tobacco products (AOR=1.60; 95% CI: 1.07–2.39; p=0.022) in contrast to adolescents lacking such knowledge. No significant difference existed between tobacco use classes and other variables.

**Table 5:** Multinomial logistic regression predicting tobacco use by sociodemographics and tobacco-related variables, a cross-sectional school-based survey, South Africa, GYTS 2011 (N=10822)

*Variables*	*Multiple tobacco vs non-tobacco*			*Combustible non-cigarette tobacco vs nontobacco*		
	*AOR*	*95% CI*	*p*	*AOR*	*95% CI*	*p*
**Sex**						
Male ®	1			1		
Female	0.65	0.49–0.87	**0.004**	0.51	0.36–0.71	**<0.001**
**Age (years)**						
≤13 ®	1			1		
14	0.44	0.19–1.03	0.057	2.27	0.78–6.62	0.130
15	1.24	0.52–2.98	0.624	2.03	0.63–6.62	0.235
16	2.22	0.88–5.61	0.090	2.16	0.58–8.11	0.250
17	2.33	0.94–5.76	0.068	2.22	0.59–8.28	0.232
≥18	4.18	1.59–10.98	**0.004**	2.44	0.65–9.11	0.182
**Grade**						
8 ®	1			1		
9	0.67	0.34–1.35	0.259	1.27	0.67–2.42	0.456
10	0.29	0.14–0.59	**0.001**	1.17	0.57–2.37	0.667
11	0.22	0.12–0.43	**<0.001**	1.23	0.57–2.67	0.598
**Tobacco advertising**						
No ®	1			1		
Yes	1.50	0.86–2.63	0.149	1.33	0.68–2.59	0.406
**School curriculum**						
No ®	1			1		
Yes	1.03	0.73–1.46	0.868	1.70	1.19–2.43	**0.004**
**Peer smoking**						
No ®	1			1		
Yes	4.07	2.93–5.66	**<0.001**	6.29	4.15–9.53	**<0.001**
**Parents/guardians smoking**						
No ®	1			1		
Yes	2.33	1.81–3.00	**<0.001**	1.91	1.31–2.79	**0.001**
**Support for smoke-free policy**						
No ®	1			1		
Yes	0.73	0.58–0.92	**0.009**	0.61	0.41–0.91	**0.016**
**Knowledge about the harm of smoking**						
No ®	1			1		
Yes	0.93	0.66–1.31	0.664	1.60	1.07–2.39	**0.022**

AOR: adjusted odds ratio. ® Reference categories.

## DISCUSSION

This study aimed to investigate patterns of tobacco use and associated factors among adolescents in South Africa using latent class analysis. The results identified three latent classes: multiple tobacco product users, combustible non-cigarette tobacco smokers, and non-tobacco users. In line with previous studies^22^, most adolescents in the sample reported non-tobacco use, leading our latent class model to categorize these individuals into the largest class, comprising 83.9% of the sample. The other remaining percentages of classes indicated current tobacco use, which were categorized as multiple tobacco product use and combustible non-cigarette tobacco products smoking. The largest of the two additional classes was multiple tobacco product use, comprising approximately 9% of adolescents. The highest response probabilities were observed for chewing tobacco, combustible cigarettes, and snuff, and high level of smoking for combustible non-cigarette tobacco products in the past 30 days. The class category labelled as combustible non-cigarette tobacco products shows that items such as cigars, little cigars, and pipes were not widely used, accounting for 3.4% of the sample.

Our findings on tobacco use among adolescents are consistent with smoking patterns observed in adult populations. Based on South African Social Attitudes Surveys from 2007 to 2018, Egbe et al.^23^ identified roll-your-own cigarettes and smokeless tobacco as the most prevalent tobacco products used after cigarettes among single product users over time. Cigarettes and waterpipes represented the predominant combination of tobacco products used by dual users. In 2010, only 0.4% of adults reported using e-cigarettes in the past 30 days, with this figure rising to 2.7% by 2018. Our results, however, did not include e-cigarette, as it was not addressed in the survey. Despite the implementation of tobacco control initiatives and the passing of South Africa’s Tobacco Products Control Act of 1993^24^, our findings indicate the ongoing prevalence of tobacco use among young individuals as previously identified in other international studies^25^. These findings indicate the necessity of enhancing tobacco control initiatives in South Africa.

Another important finding from this study was the underlying pattern of tobacco use by sex and age. Sex differences were prominent across classes. The class of non-tobacco users predominantly included females, whereas the classes of multiple tobacco product users and combustible non-cigarette tobacco products smokers had a higher percentage of males. The finding that males are more likely than females to use multiple tobacco products aligns with several studies in tobacco research and public health^26^. This may adhere to the established stages of the cigarette smoking epidemic, as earlier stages indicated that males had higher prevalence rates of cigarette smoking than females^27^. However, in our study, the male and female differences in multiple tobacco product use is not too wide and corresponds to a 2:1 ratio. It is possible that the trend of multiple tobacco product use might set the stage for a new tobacco epidemic in South Africa, where females could eventually reach use rates similar to those of males. In addition, our results revealed that older adolescents (≥18 years) were positively associated with multiple tobacco product use. This aligns with the 2016 South Africa Demographic and Health Survey (SADHS) findings, which indicated a high prevalence of smoking among individuals aged 15–24 years for both males and females, consistent with other previous studies^28^.

In this study, the odds ratio based on multinomial logistic regression analysis revealed a strong association between peer smoking, multiple tobacco product use and combustible non-cigarette tobacco smoking. Adolescents exposed to peer smoking were four times more likely to use multiple tobacco products and six times more likely to smoke combustible non-cigarette tobacco products (cigars, little cigars, and pipes). Our findings highlight the significance of social influences on the use of multiple tobacco products. Notably, adolescents who use multiple tobacco products seem to be at highest risk of associating with friends who also use multiple tobacco products^29^. Peer influences and normative beliefs are acknowledged as factors within the social environment that contribute to the initiation and maintenance of tobacco smoking^30^. Insufficient evidence indicates that peer smoking of combustible non-cigarette tobacco products significantly predicts adolescents’ use of alternative tobacco products, including e-cigarettes^31^. Consequently, the smoking of combustible non-cigarette tobacco products by peers may contribute to multiple tobacco product use, especially as certain products (such as waterpipes and e-cigarettes) are regarded as social and interactive products used during leisure time with friends and family^32^. A complex facet of human behavior, the social influence of peers and family, is not addressed in tobacco-related interventions.

Adolescents who supported smoke-free policies had lower odds of using multiple tobacco products and combustible non-cigarette tobacco products compared to those who did not support such policies. This association highlights the potential protective role that support for tobacco control measures can play in reducing tobacco use among young people. Adolescents who endorse smoke-free environments may be more health-conscious, better informed about the harms of tobacco use, or more influenced by anti-smoking norms within their families, schools, or communities^33^. Their support could also reflect a broader alignment with public health values and a greater susceptibility to tobacco prevention messaging. This finding aligns with previous research demonstrating widespread and increasing adolescent support for smoke-free environments. Studies have consistently shown that young people generally favor restrictions on smoking in public places such as schools, parks, restaurants, and public transportation^34^. A systematic review and meta-analysis, examined 12 studies assessing levels of support for smoke-free policies both before and after their implementation. Their review revealed a consistent increase in support post-implementation, suggesting that such policies may not only be accepted but embraced by adolescents once they are in place. This growing support could be due to increased awareness of secondhand smoke risks, shifts in social norms, or positive experiences in smoke-free environments.

In addition, the findings from this study provide evidence that adolescents who use multiple tobacco products and combustible non-cigarette tobacco products possess knowledge of tobacco harmful effects to smokers and non-smokers through secondhand smoking. Consequently, enhancing adolescents’ awareness of the negative effects of tobacco use would increase both their intentions to quit^35^ and their support for and adherence to smoke-free policies. This may serve as motivation for the mobilization of actions against tobacco use in South Africa, as previously done at the international level^36^. Therefore, preventive measures and the reinforcement of regulations are essential in addressing the use of multiple tobacco products and non-cigarette tobacco products among adolescents. A comprehensive analysis of diverse product combinations by both sex and their contributions to the use of multiple tobacco products and non-cigarette tobacco products, would yield valuable insights with significant implications for tobacco control in South Africa. Policy modifications may also be guided by the latent classes identified in this study.

### Limitations

This study presents several limitations. First, the data were collected in 2011, which may not fully reflect current patterns of tobacco use among adolescents in South Africa, particularly given the emergence of new tobacco products and shifts in tobacco control policies over time. Second, the study employed a cross-sectional design, which precludes any inference of causality between the identified correlates and patterns of tobacco use. Longitudinal studies are needed to assess changes in tobacco use behaviors over time and establish temporal relationships. Third, the sample was restricted to school-going students who were present on the day the survey was administered, potentially introducing sampling bias. Adolescents who had dropped out of school or were absent during data collection were excluded, limiting the generalizability of the findings to the broader adolescent population. Additionally, private schools were underrepresented in the sampling frame, which may skew the findings, as tobacco use behaviors could differ by school type and socio-economic context. Fourth, the GYTS data did not investigate alternative tobacco products, such as e-cigarettes, waterpipes, and roll-your-own products, which may be associated with patterns of tobacco use among adolescents. Lastly, in latent class analysis, adolescents are categorized probabilistically. Consequently, this study may account for classification uncertainty, as adolescents may not fall perfectly into a single class.

## CONCLUSIONS

The use of multiple tobacco products poses significant risks for adults and is even more concerning for adolescents. A better understanding of adolescents who use multiple tobacco products is essential to guide interventions aimed at this group. This study expands the literature on adolescent tobacco use by providing unique evidence concerning the use of multiple tobacco products, non-cigarette tobacco smoking, and various tobacco-related factors using different classes of adolescent tobacco use, while suggesting implications for future research and policy development. Three classes emerged from the data, revealing distinct patterns of tobacco use that may have different risk profiles and responsiveness to tobacco control programs. Policy interventions are progressively tailored, therefore, grouping adolescents based on use patterns may facilitate the development of more specific use profiles and the formulation of effective interventions. Targeted cessation interventions should be explored to assist adolescents who use multiple tobacco products. Adolescents should be educated about the dangers of using multiple tobacco products and other forms of tobacco, while also being safeguarded from social, peer, and industry pressures to mitigate the risk of using multiple tobacco products.

## Data Availability

The data supporting this research are available from the GYTS repository: https://extranet.who.int/ncdsmicrodata/index.php/catalog/175/download/1860
